# Myocardial Ischemic Syndromes, Heart Failure Syndromes, Electrocardiographic Abnormalities, Arrhythmic Syndromes and Angiographic Diagnosis of Coronary Artery Spasm: Literature Review

**DOI:** 10.7150/ijms.43472

**Published:** 2020-04-27

**Authors:** Ming-Yow Hung, Nicholas G. Kounis, Meng-Ying Lu, Patrick Hu

**Affiliations:** 1 Division of Cardiology, Department of Internal Medicine, Shuang Ho Hospital, Taipei Medical University, New Taipei City, Taiwan; 2 Taipei Heart Institute, Taipei Medical University, Taipei, Taiwan; 3 Division of Cardiology, Department of Internal Medicine, School of Medicine, College of Medicine, Taipei Medical University, Taipei, Taiwan; 4 Department of Cardiology, University of Patras Medical School, Rion, Patras, Achaia, Greece; 5 University of California, Riverside, Riverside, California, USA; 6 Department of Cardiology, Riverside Medical Clinic, Riverside, California, USA

**Keywords:** myocardial ischemic syndrome, heart failure syndrome, arrhythmic syndrome, provocative testing, coronary artery spasm

## Abstract

In coronary artery spasm (CAS), an excess coronary vasoconstriction causing total or subtotal vessel occlusion could lead to syncope, heart failure syndromes, arrhythmic syndromes, and myocardial ischemic syndromes including asymptomatic myocardial ischemia, stable and unstable angina, acute myocardial infarction, and sudden cardiac death. Although the clinical significance of CAS has been underrated because of the frequent absence of symptoms, affected patients appear to be at higher risk of syncope, serious arrhythmias, and sudden death than those with classic Heberden's angina pectoris. Therefore, a prompt diagnosis has important therapeutic implications, and is needed to avoid CAS-related complications. While a definitive diagnosis is based mainly on coronary angiography and provocative testing, clinical features may help guide decision-making. We perform a literature review to assess the past and current state of knowledge regarding the clinical features, electrocardiographic abnormalities and angiographic diagnosis of CAS, while a discussion of mechanisms is beyond the scope of this review.

## Introduction

Angina pectoris is caused by a transient imbalance between myocardial demand and supply 1, 2, which results in 2 types of ischemia, non-exertional supply ischemia and exertional demand ischemia 3. Furthermore, coronary lesions are dynamic 4. As a result, non-obstructive coronary lesions might restrict increases in coronary blood supply if vascular tone is augmented 4, and hence myocardial ischemia is not always preceded by increased oxygen demand 5. Among the mechanisms of angina pectoris, coronary artery spasm (CAS) had long been considered the chief one 6, albeit as yet unproved until 1940s when the recognition of angina-associated fixed atherosclerotic obstruction post mortem led to a revision of the theory that CAS may produce paroxysmal myocardial ischemia 7-9 (Table 1).

However, Dr. Myron Prinzmetal (1908-1987) published his observations on "A variant form of angina pectoris" in 1959, which was the 1^st^ article 10 distinguishing it as a distinct entity from the classic angina pectoris (pectoris dolor) described by Dr. William Heberden (1710-1801) based on 20 cases with this affliction in 1772 11, which occurred when increased cardiac work or emotional disturbance provoked chest pain and was relieved by rest or the administration of nitroglycerin. Although some patients with non-exertional angina of Heberden's cases probably represents the 1^st^ reported variant form of angina pectoris, his description of non-exertional angina is vague. On the other hand, patients with Heberden's angina may also manifest CAS 12.

In Prinzmetal's 1^st^ report of 32 cases of variant angina, of which 20 were personally observed and 12 were from the literature, the pain associated with transient non-progressive ST-segment elevation appeared at rest or during ordinary activity but was not brought on by exercise or emotional disturbance. Among the 32 patients studied, 12 developed myocardial infarction during follow-up 10. Because coronary atherosclerosis was a common finding in both forms of angina pectoris post mortem, and the attack usually occurred with the subject at rest, when vascular hypertonic activity is physiologically greatest 10, vascular hypertonus proposed by Prinzmetal et al. or CAS proposed by other researchers was the explanation for variant angina. Although CAS had never been proved 7,13 within a decade following the 1^st^ report of coronary angiography in 1959 14, CAS was documented angiographically in early 1970s in patients of variant angina 13, 15, 16.

In the 1970s and 1980s, the diagnosis of CAS by coronary angiography in the catheterization laboratory was not rare. It then became clear that CAS could occur in patient with atherosclerotic obstructive coronary artery disease 10, 11 or angiographically normal coronary arteries, which was referred to as “variant of the variant” 15 or “coronary vasospastic angina” 17. Moreover, ST-segment depression rather than non-progressive elevation occurred more commonly in CAS 18, 19. Therefore, the term “variant angina” is used for CAS-related angina with transient non-progressive ST-segment elevation. Additionally, CAS and acute coronary events can be caused by allergic reactions, with mediators released during mast cell degranution such as histamine, chymase, leukotrienes, platelet activating factor acting on coronary vascular smooth muscle cells that constitute the pathophysiologic basis of Kounis syndrome 20, 21. Collectively, the presence of atherosclerotic obstructive coronary artery disease cannot be considered as the only determinant of angina pectoris 22.

## Prevalence

CAS-related angina and myocardial ischemia, regardless of symptoms, are common 10, 23, though not nearly as common as classic Heberden's angina pectoris. The prevalence of CAS and obstructive coronary artery disease in Taiwanese general population during a follow-up period of 12 years is 0.067% and 8.7%, respectively 24. However, the prevalence of CAS in different general populations remains to be defined. On the other hand, the number of CAS patients would rise among angina patients when systematic rules for its detection are applied 23. Therefore, undoubtedly the incidence of CAS-related myocardial ischemia could be significantly greater than is indicated by the demonstration of variant angina if one considers that (1) angina at rest with S-T segment depression and with pseudonormalization of T waves also appears to be caused by CAS; (2) that asymptomatic ischemic episodes are frequent 23, 25; and (3) that cold-induced angina may result from CAS 26.

There are wide differences in the CAS prevalence in different countries. The frequency of CAS in Japan is greater than that in western countries 27 and the diagnosis of variant angina with transient non-progressive ST-segment elevation among patients with angina referred to Japanese medical institutions is as high as 40% 17. In addition, multiple spasms (≥2 spastic coronary arteries) induced by provocative testing in Japanese (24.3%) 28 and Taiwanese populations (19.3%) 29 occur more frequently than in Caucasians (7.5%) 30. CAS is more prevalent among men than women in East Asia as well as Western countries 17, 29. Most CAS occur in patients between 40 and 70 years of age and the prevalence decreases after 70 years of age 10, 17, 29. Previous Asian and German studies have shown that the prevalence of CAS is around 50% in patients with angina and 57% in Asian patients with acute coronary syndrome without obstructive coronary artery disease 31-33. Among provocative vasomotor studies in patients with acute coronary syndrome without a culprit lesion, 79% of Japanese patients had a positive result 34, whereas 16% of French 35 and 49% of similar German 33 patients developed CAS after intracoronary acetylcholine. On the other hand, CAS can be difficult to diagnose owing to premedication with spasmolytic drugs such as nitroglycerin or calcium channel blockers, avoidance of coronary constrictors, and variation of disease activity. The prevalence of CAS tends currently to decrease, especially in Japan, for many reasons such as less performing vasoreactivity test (time consuming), or widespread use of statins and calcium channel blockers 36. Taken together, these results suggest that while racial differences exist in coronary vasomotor response 27, CAS prevalence in different populations is as yet unknown.

## Myocardial Ischemic Syndromes with or without Symptoms

Symptoms vary widely and may be silent (Table 2), among which the length (longer duration) of CAS is an essential determinant. The typical CAS-related angina at rest is a vague sensation of compression in the precordium or upper abdomen 17 with radiation to the neck, jaws, left shoulder, and elsewhere 17. The angina may be accompanied by cold sweats and disturbance of consciousness including syncope 17. During CAS attack, a lowering of blood pressure or decrease in pulse pressure may appear 10. Although prolonged episodes of CAS can lead to angina and even myocardial infarction 37, brief episodes of CAS without any symptoms can result in silent myocardial ischemia, or cause life-threatening arrhythmias, resulting in sudden death 38-39. The incidence of silent myocardial ischemia is more than 2 times higher than that of symptomatic ischemia 38. Hence, there is a wide spectrum of CAS-related myocardial ischemic syndromes, including silent myocardial ischemia, stable angina, unstable angina, acute myocardial infarction and sudden death 23, 37, 38, 40. In addition, CAS has been reported to be associated with migraine syndrome 41, Raynaud's phenomenon 41 and spasm of digital arteries 42.

CAS occurs mostly at rest or at night, sometimes in association with awakening from sleep 43, especially from midnight to early morning 10, 23, 38-40, 44-46, during which 67% of CAS attacks are asymptomatic 17. A previous report using intravenous methergine provocation tests showed that the frequency of CAS was 38% when angina occurred only at rest, compared with 13.8% when angina at rest and during exertion 30. Moreover, CAS precipitated by variable threshold effort, particularly in the morning in some patients 39, can be associated with ST-segment depression or elevation, or pseudonormalization of T waves 18, 23, suggesting that spastic arteries are abnormal because normal coronary arteries dilate during exercise.

The incidence of CAS shows daily, weekly, monthly, and circadian variations 47. The complexity of the neural networks modulating the tone of the coronary arteries 48, and the association of the occurrence of CAS in the early morning with rapid eye movement 49, during which time there is a rapid elevation of sympathetic activity, suggest that changes in the activity of the autonomic nervous system may be involved in the circadian variation of CAS. With the use of coronary artery angiography in patients with CAS, Yasue at al. 46 found that in the early morning, the tone of the major coronary artery was increased and its diameter was smaller than normal, while in the afternoon, the major coronary artery was usually dilated. Therefore, most patients with CAS have a diurnal variation in the exercise capacity. Of note, as attacks of CAS may occur frequently, ie, several times every day, or may not occur for several months to several years 17, patients should be followed up closely.

## Heart Failure Syndromes

CAS, especially multiple spasms 50, causes myocardial necrosis (a form of reperfusion injury) 51, which leads to a reduction in diastolic compliance during angina 52 and heart failure with reduced ejection fraction (HFrEF) 50, 53-56 (Table 2). It has been shown that the left ventricular dysfunction may return in about 2 minutes to basal levels as the electrocardiographic changes start reverting to the pre-CAS state 22, 57. On the other hand, about 30-47.6% of hospitalized patients with optimally treated HFrEF <45% has provoked CAS 50, 53-55. While the prevalence of hypertension and smoking was higher in CAS-related than non-CAS-related HFrEF in a previous study 55, further studies are needed to identify the risk factors of CAS-related HFrEF. The CAS-related regional wall motion abnormality, left ventricular dilation and reduced ejection fraction improve 6 months to >1 year after medical treatment for CAS, including calcium channel blockers and nitrate/nicorandil 50, 53. HFrEF, such as dilated cardiomyopathy in Syrian hamsters 51, 58 and in German patients 59, with microcirculatory disorders which is possibly due to CAS, can be improved by treatment with verapamil and diltiazem, respectively, through vasodilator effect. Notably, while adjunctive diltiazem treatment in possibly CAS-related dilated cardiomyopathy has beneficial effects on mortality, hemodynamics and symptoms through reducing afterload, decreasing arrhythmias, and inhibiting catecholamines 59, diltiazem in patients with HFrEF due to infarction has a poor outcome 60. On the other hand, the use of calcium channel blockers in patients with non-ischemic HFrEF is not a first-line therapy at the present time. Therefore, although 1st generation dihydropyridine and nondihydropyridine calcium channel blockers (except amlodipine and felodipine) should generally be avoided in patients with non-CAS-related HFrEF since they provide no functional or mortality benefit and may worsen outcomes 61, if patients with HFrEF have provoked CAS, the use of calcium channel blockers might be beneficial to improve CAS-related myocardial ischemia 50, 54. Future studies are required to elucidate the potential treatment role of calcium channel blockers in CAS-related HFrEF. On the contrary, the use of β-blockers in patients with CAS and HFrEF may result in the aggravation of CAS 56.

In addition, the prevalence of atrial fibrillation in the patients with dilated cardiomyopathy and CAS is greater than that in dilated cardiomyopathy without CAS [67% vs 8% (P<0.05)] 54. Hence, dilated cardiomyopathy with atrial fibrillation may be a clue for identifying CAS 54. Taken together, although there is no guideline addressing the role of calcium channel blockers in CAS-related heart failure 55, CAS should be considered in the differential diagnosis of dilated cardiomyopathy or HFrEF as calcium channel blockers may be a promising therapy 53, 55, and provocative tests for CAS can be safely performed after the stabilization of heart failure 56.

## Electrocardiographic Abnormalities and Arrhythmic Syndromes

All CAS-related electrocardiographic changes may occur without the subsequent angina 22 (Table 2), as angina develops in only 20-30% of episodes of ischemic ST-segment changes 17. There is a great variability of the electrocardiographic changes in the same patient during different CAS episodes 22, because CAS may occlude ≥1 vessel and may diffusely involve smaller arterial branches, resulting in myocardial ischemia with ST-segment elevation or depression or with only T wave changes 23. Occasionally, the ST-segment changes during CAS may seem to improve a previously depressed ST-segment 10, 47. In Prinzmetal's original report, spurious improvement of the prior low T wave during angina was noted 10. It has been demonstrated that the pseudonormalization of T waves, initially described in 1970s when continuous electrocardiographic recordings became available 62, is directly related to transmural myocardial ischemia in CAS 23. Therefore, an electrocardiography showing “improvement”, or pseudonormalization, of ST-segment and T wave changes should be studied carefully for this may be the only graphic change of CAS 10.

On the other hand, a normal electrocardiography does not rule out the presence of CAS 10, as it may be recorded when the CAS starts early or when the CAS is only mild 47. CAS of a major coronary artery results in ST-segment elevation in the leads corresponding to the distribution of a large coronary artery and the subsequent locations of myocardial infarction 10. Of note, thallium scintigraphy revealed a regional massive and localized reduction of myocardial perfusion during S-T segment elevation and pseudonormalization of T waves 23. While a transmural ischemia and injury results in ST-segment elevation, peaking of T waves, or pseudonormalization of T wave changes, a less severe, non-transmural subendocardial myocardial ischemia results in ST-segment depression or T wave inversion 22. Notably, CAS is associated more frequently with ST-segment depression rather than ST-segment elevation 18, 19, 63. ST-segment depression appears when CAS of a major artery is less severe, when a major artery receiving collaterals is completely occluded, or when a small artery is completely occluded 64. This condition may exist in unstable angina/non-ST-elevation myocardial infarction. About 45% of patients with angina at rest and ST-segment depression alone has CAS 30. Moreover, ischemic episodes characterized by ST-segment elevation or depression or by T wave change (inversion, peaking, pseudonormalization of negative wave) may occur in the same patient with CAS within a few minutes 22, 23. In addition to ST-segment changes, a taller and broader R wave 10, 65, a decrease in magnitude of S wave, peak T wave and negative U wave may also appear 47. Because the location of CAS may be fixed, or fluctuate from one vessel to another over time 66, ST-segment elevation and depression could occur alternatively in the same patient or even in the same lead within minutes or hours 23. Furthermore, electrocardiographic changes may vary during repeated provocative tests and recurrent spontaneous attacks 67, 68. Hence, the appearance of ST-segment changes may differ over time.

The ST-segment and T wave changes of CAS are associated with the subsequent left ventricular dysfunction and practically every known form of arrhythmia 10, 32, 47, 69-73, sometimes life-threatening 22, 58, which often occur in patients with acute coronary syndrome 32. The high incidence of arrhythmia in CAS may be related to the usually severe ischemia or to the sudden massive reperfusion 69. Among arrhythmias, ventricular premature complex was most common and high-grade atrioventricular block was next most common 11, and of special importance are sinus node arrest and ventricular fibrillation (VF) 69. Bradycardia and supra-His conduction disorders tend to occur preferentially during inferior wall ischemia, usually indicating the most commonly CAS-involved right coronary artery, given the junctional location of the block due to ischemia of the branch supplying the sinoarterial and atrioventricular node 74, while an infra-His block may occur when CAS involves left coronary arteries 75. As a result, sinus bradycardia occurs more frequently than sinus tachycardia, and when profound sinus bradycardia and periods of sinoarterial block or sinus arrest occur, it may cause an acute form of the sick sinus syndrome complicated by syncope 69. In addition, right bundle branch block 76 and intermittent left bundle branch block 11, 12, 43, 77 have been reported to be associated with CAS. CAS-related sudden death most frequently results from bradyarrhythmias, rather than from tachyarrhythmias 78, 79. Nitrates and calcium channel blockers may be effective in the control of CAS induced arrhythmia, but other traditional antiarrhythmic agents and pacemaker treatment may be required in some patients 69.

Arrhythmias, particularly ventricular, appear more frequently through unknown mechanisms during CAS attacks in >50% of cases than during attacks of classic Heberden's angina pectoris 10, 43 (Table 2). Ventricular arrhythmias are more common during anterior wall ischemia 74. Sudden death with normal appearing coronary arteries on autopsy examination has been attributed to VF complicating CAS 80. Although VF uisually needs to be terminated by cardioversion 30, CAS-related VF rarely reverts spontaneously 81-83. In addition, VF was found to be asymptomatic in 43% and nonsustained in 40% episodes in a study of patients with implantable cardioverter defibrillators 84. The incidence of syncope or pre-syncope is 25% when VF is <10 seconds, compared with 62% if VF is ≥10 seconds 84. Therefore, CAS should be considered in the differential diagnosis of syncope.

On the other hand, severe CAS may cause fatal pulseless electrical activity or asystole without complications of ventricular tachycardia or VF 85-88. Triple-vessel severe CAS can cause the heart to suddenly stop beating due to pulseless electrical activity and flash-freeze the entire myocardium in an instant, resulting in unrecognized coronary flow 88. Consequently, contrast medium may stay in the coronary arteries for a prolonged period of time despite intracoronary administration of nitroglycerine. Prolonged continuous cardiac massage has been effective for resolving CAS-related pulseless electrical activity 88. However, cardiac pacing or implantable cardioverter defibrillator might not restore frozen myocardium to viable muscle during pulseless electrical activity arrest, and may lead to unexplained death after the implantation 85, 88. Furthermore, CAS-related ischemia of the sinus node artery or atrioventricular node artery can influence the occurrence of pulseless electrical activity or asystole 88. Collectively, CAS may cause pulseless electrical activity or asystole without ventricular arrhythmias.

## Diagnosis

### Differential Diagnosis

**Figure 1:**
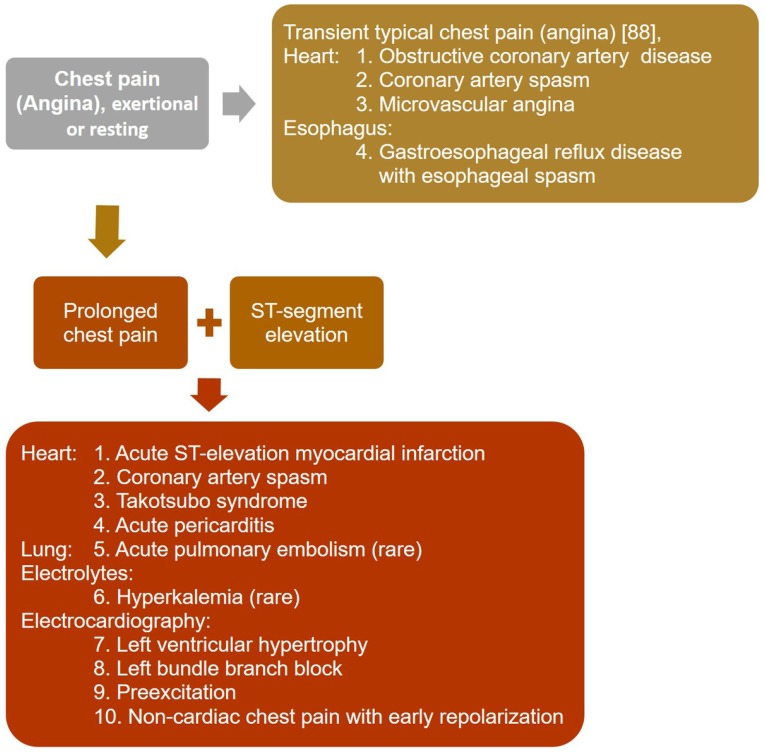
Diagnostic approach to patients presenting with transient typical chest pain, regardless of electrocardiographic ischemic changes, and prolonged chest pain with ST-segment elevation.

For patients presenting with transient typical chest pain 89 with exertion or at rest, regardless of electrocardiographic ischemic changes, the diagnostic approach should include obstructive coronary artery disease (Figure 1). Other diagnosis that should be considered include gastroesophageal reflux disease with esophageal spasm 90 and microvascular angina, which has largely replaced cardiac syndrome X 91.

When ST-segment elevation appears, the diagnoses most often confused with CAS, which presents with transient non-progressive ST-segment elevation, include acute ST-elevation myocardial infarction, acute pericarditis, left ventricular hypertrophy, left bundle branch block, preexcitation, non-cardiac chest pain associated with early repolarization 92, takotsubo syndrome 93, acute pulmonary embolism 94, and hyperkalemia 95 (Figure 1). Patients with acute ST-elevation myocardial infarction usually present with pain and ST-elevation for >15 minutes, which are not resolved by acute therapy with nitroglycerin or calcium channel blockers 96. Acute pericarditis 97 and takotsubo syndrome 93 are characterized by repetitive episodes of chest pain for hours or days over a period of weeks or months. The chest pain in acute pericarditis is generally worse when lying supine and relieved by sitting, and might radiate to the neck, arms, or left shoulder, making differentiation from myocardial ischemia difficult 97.

### Diagnostic Coronary Angiography

Although the key to the clinical diagnosis in medicine is the taking of a history, CAS may present with or without symptoms 38, occasionally relieved by exertion 10. Furthermore, CAS can exhibit either normal electrocardiographic findings at the beginning of an attack or when the attack is mild 47, or ST-segment elevation or depression during the attack 64, hence a diagnosis of CAS cannot be directly made by symptoms 30. Standard 12-lead electrocardiography 64, ambulatory electrocardiography 47, or exercise stress tests 10, 98. During the monitoring period of ambulatory electrocardiography, the attack may not appear 47; however, brief episodes of ST-segment changes, heart blocks or potentially fatal ventricular arrhythmias may be detected (Table 2), which may allow for characterization of the attacks. Hence, ambulatory (holter) electrocardiography monitoring should be performed for patients in whom the diagnosis of CAS is being considered. Although a patient may have CAS if ST-segment elevation or depression of ≥0.1 mV in at least 2 contiguous leads, pseudonormalization of T waves 23, or new negative U waves during exercise stress tests 17, the exercise testing results are usually negative in CAS 98. Collectively, it is reasonable to refer a patient with suspected CAS for coronary angiography even if the stress test and ambulatory monitors are normal, particularly if episodes of pain do not occur during ambulatory monitoring.

A recently developed clinical diagnostic score for prediction of CAS in patients with acute chest pain consists of 6 factors, including allergies, asthma, angina attack at rest, ST-segment elevation, myocardial bridge, and hyperventilation test 99. However, myocardial bridge is present anatomically in approximately 25% of patients based on autopsy and computed tomography, but only results in angiographically detectable systolic compression in less than 10% of patients 100. Therefore, a larger scale of clinical data is needed to examine its predictive accuracy and specificity.

Coronary angiography with provocative testing, initially described in 1972 101, is the only certain method of diagnosing CAS 102. Although an occasional angiographic documentation of CAS in a patient with angina and normal electrocardiography at rest was reported by Gensini et al. in 1962 103, it was not until the early 1970s that the hypothesis of CAS was provided by serial reports. While the 1^st^ CAS, probably catheter- or contrast- related, with reproduced chest pain was demonstrated angiographically in a patient with Prinzmetal angina in 1972 83, spontaneously occurring CAS was not proved until 1973 when Prinzmetal angina of a non-smoking woman was demonstrated by coronary angiography 13. The 1^st^ provocative testing using intravenous and intracoronary ergonovine were performed by the Cleveland Clinic in 1972 101 and Hackett et al. in 1987 104, respectively. Interestingly, Yasue et al. reported that the use of subcutaneous injection of methacholine induced CAS in 1974 105, and the usefulness of intracoronary acetylcholine in 1988 106.

In patients with ST-segment elevation during episodes of angina and a normal coronary angiography, provocative tests usually are not necessary for diagnosis of CAS 102. Provoked CAS is defined as a reduction of >50% 102, >70% 106, >75% 54, 101, 107, 108, >90% 17, 55, 108, 109, or 99-100% 53 in luminal diameter with or without symptoms and/or ischemic ST-segment changes compared with postintracoronary nitroglycerin.

The diagnostic criteria of positive provocative testing for CAS was initially described by Chahine et al. according to 12, 30: (1) appearance of 100% occlusion of a segment of coronary artery; (2) appearance of significant narrowing (≥75%) of a segment of coronary artery; (3) the disappearance, either spontaneous or by administration of nitroglycerin, of the ≥75% narrowing or total occlusion. In humans with one-vessel obstructive coronary artery disease, basal resting myocardial blood flow remains constant regardless of the severity of coronary artery obstruction 110. During hyperemia as in exertion, the ability to increase myocardial blood flow becomes impaired with obstruction >50% and is virtually abolished at >70% 111. In patients with one-vessel obstructive coronary artery disease, a percent diameter stenosis of ≥50% and a percent area stenosis of ≥75% correspond to haemodynamically significant stenosis as evaluated by perfusion images 111. Animal experiments indicate that only in the presence of a ≥90% acute lumen reduction does flow through the stenosis decrease below resting level 112. CAS has been observed in obstructive coronary artery stenosis varying from 50% to subocclusive 22; it is possible that a small reduction in luminal diasmeter, not easily detectable angiographically, may suffice to reduce the flow in these cases 22. Therefore, Yasue et al. 47 suggested no limits on the reduction in luminal diameter for CAS diagnosis since myocardial ischemia results from the changes of vessel size. Individual patients have their own specific clinical features; hence, decisions regarding diagnosis of CAS should be based on the specific conditions of patients 17. Collectively, because CAS can occur with variable threshold exertional angina and rarely with exertional angina only, a reduction of >50% to 100% in luminal diameter with or without symptoms and/or ischemic ST-segment changes compared with postintracoronary nitroglycerin should be considered a positive test for CAS.

The severity and location of CAS and the associated electrocardiographic abnormalities have been variable in different episodes of CAS 13, 22, 63. However, among the spasm-provoked arteries, the right coronary artery and left circumflex artery are the most and least frequently involved, respectively 12. The susceptibility of the right coronary artery to spasm is demonstrated by the common occurrence of proximal spasm induced by the catheter tip during angiography 43.

During provocative tests, the occurrence of severe chest pain with ST-segment depression but without epicardial CAS has been termed microvascular CAS 108. While a substantial proportion of CAS patients with angina involving the epicardial coronary arteries also have microvascular CAS 113, coronary microvascular dysfunction is a common finding in invasively managed angina patients with non-obstructive 114 or obstructive epicardial coronary artery disease 115. The current gold standard for clinically invasive assessment of microvascular function in response to metabolic demands has been coronary flow reserve, which remains normal until diameter stenosis of epicardial large artery is >50% 116, 117. In addition, microvascular CAS can be inferred when coronary slow phenomenon, defined as TIMI frame count >25, appears 114. Hence, in patients with non-obstructive or obstructive epicardial coronary artery disease, microvascular CAS may be considered one of the contributing factors for ischemia-like symptoms and myocardial ischemia. The involvement of the microvascular CAS adds a new diagnostic and therapeutic dimension to the problem, which needs to be considered in future studies, as coronary microvessels do not necessarily respond to currently used vasodilator agents in an identical fashion as epicardial coronary arteries.

The provocative testing at present involves the use of ergonovine or acetylcholine 102. Ergonovine, an ergot alkaloid used to control postpartum uterine bleeding, was found in 1949 to provoke angina, and was proposed in 1963 as a diagnostic test for coronary disease 118. Although methylergonovine, structurally similar to norepinephrine, and acetylcholine stimulate endothelial nitric oxide generation 119, they cause smooth muscle cell contraction in the setting of endothelial dysfunction 120-123. In normal coronary arteries, only mild generalized vasoconstriction (<20% diameter reduction) would be induced 115, 124. Ergonovine testing in the catheterization laboratory was used in the late1970s and early 1980s to help identify the mechanism of chest pain when non-obstructive coronary artery disease was found by angiography. In early studies, however, patients receiving very high doses of intravenous ergonovine frequently had severe angina and a reported death in a small series caused the intravenous route to be abandoned 125. The frequency of provoked CAS by the intracoronary administration is about 2.5-fold higher than that by the intravenous administration of ergonovine and acetylcholine 28, 30; however, there is no difference concerning the incidence of provoked CAS between ergonovine and acetylcholine 126. While provoked CAS by ergonovine tends to be proximal and focal, CAS provoked by acetylcholine is distal and diffuse 127-129. Although the intracoronary injection of ergonovine and acetylcholine provoked CAS in 65% and 80% in a previous study 128, respectively, no differences existed regarding the provoked CAS between intracoronary ergonovine and acetylcholine in a later study 129. Further studies are needed to evaluate the coronary response between the ergonovine and acetylcholine examinations.

To ensure a valid diagnosis, vasodilators (calcium antagonists and nitrates) must be withdrawn for ≥24 hours except for sublingual nitroglycerin if necessary 17, 102, 130. Prophylactic administration of coronary vasodilators at the beginning of coronary angiography should be avoided 12. On the other hand, the nitroglycerin or atropine 131 solution for intracoronary administration must be well prepared before starting provocative testing to abolish documented CAS immediately. The intracoronary administration of methylergonovine affords the opportunity to evaluate the left and right coronary arteries separately with small dosing increments of 5 to 10 μg and a total dose not to exceed 50 μg 102. The effectiveness of intracoronary administration of acetylcholine in doses of 10 to 100 μg is comparable to methylergonovine 102, 132, 133. Cautious administration of increasing doses of intracoronary ergonovine was given, with angiography several minutes after each dose or until whenever chest pain or electrocardiographic changes occurred. During coronary angiography, 2 sets of coronary cineangiograms are performed before and after the administration of nitroglycerin 12.

About one-third of patients with dilated cardiomyopathy has CAS and multiple episodes and locations of CAS may lead to HFrEF 50. To help determine the causes of HFrEF for patients with no fixed coronary artery obstruction, the spasm provocative testing should be performed with careful attention 126, 134 as it has a potential to cause angina, hypotension and various arrhythmias. For intracoronary acetylcholine provocative testing, 100 μg acetylcholine is injected directly into the left and right coronary arteries separately 53. To provoke CAS by ergonovine (methylergometrine maleate), ergonovine was injected in incremental doses of 1, 5, 10, and 30 μg into the left and 1, 5, and 10 μg into the right coronary artery over 1 minute with a 3-minute interval between each injection 54. In previous Japanese studies 50, 55, acetylcholine was injected in incremental doses of 20, 50, and 100 μg in 5-10 ml 0.9% saline solution into the left and of 20, 50 50, 56, and 80 55 μg into the right coronary arteries over 20 seconds, On the other hand, ergonovinie in 0.9% saline solution was injected in total doses of 40 μg/40ml into the RCA and of 64 μg/64ml into the LCA over 4 min each 50. Although severe HFrEF was previously considered an absolute contraindication 102, CAS provocative testing has been safely performed recently 50, 53-55.

While the complications of intracoronary provocative testing include angina, hypotension, dyspnea, flushing, nausea, vomiting and various arrhythmias 47, 135, systemic effects, such as hypertension, are avoided 136. No procedure-related mortality, myocardial infarction or irreversible complications have been reported during intracoronary provocative testing 28, 123. Because VF is a possible complication following intracoronary provocative testing, its use inside the cardiac catheterization laboratory is highly recommended. The absolute contraindications to methylergonovine include high-grade left main coronary artery disease, severe left ventricular dysfunction, moderate to severe aortic stenosis, severe hypertension and pregnancy 102. A multicenter, retrospective study showed that the rates of serious cardiac complications were <1% in patients undergoing a pharmacological provocative testing 137. However, acetylcholine provocative testing were associated with a higher rate of serious cardiac complications than ergonovine tests (odds ratio: 1.75, 95% confidence interval: 1.13‐2.69, P = 0.011) 137.

## Conclusion

The management of angina should not be oversimplified as CAS not only induces angina but also causes HFrEF. Patients with HFrEF and CAS may have associated atrial fibrillation. In addition, asymptomatic CAS patients could die suddenly of VF, pulseless electrical activity or asystole. Awareness of CAS is important for clinicians for appropriate management, and preventing progression to more severe atrioventricular block necessitating permanent pacemaker implant. Furthermore, since the coronary vasoconstriction is nonspecific and the angina of CAS is not relieved by rest, CAS-related angina in the same patient may occur under different conditions. As a result, it is important to identify patients with CAS because treatment strategies are different between obstructive coronary artery disease and CAS, and the administration of calcium channel blockers is necessary to improve left ventricular function for CAS-relarted HFrEF.

## Figures and Tables

**Table 1:** Evolution of clinical Importance of CAS.

Year	Event
17^th^ century	Edward Hyde (1609-1674), a nonmedical writer and historian, describes his father's ailment as the 1^st^ case of chest pain syndrome, which is very suggestive of angina pectoris, along with complete follow-up on the disease until his father's sudden death in 1632, in his biography, Life of Edward, Earl of Clarendon 138.
1772	The 1^st^ account of classic angina pectoris is described by William Heberden (1710-1801) 11. It has 8 characteristics 138, The access and the recess of the fit is sudden. There are long intervals of perfect health. Wine, and spirituous liquors, and opium afford considerable relief. It is increased by disturbance of the mind It continues many years without any other injury to the health. In the beginning it is not brought on by riding on horseback, as in a carriage, as is usual in diseases arising from scirrhus or inflammation. During the fit the pulse is not quickened Its attacks are often after the 1^st^ sleep, which is a circumstance common to many spasmodic disorders
1895	The 1^st^ practical electrocardiography is invented by Dr. Willem Einthoven 139.
1940	Among the mechanisms of angina pectoris, although CAS has long been considered the chief one 6, the difficulty of visualizing CAS of the arteriosclerotic arteries in patients with angina pectoris is frequently pointed out 7.
1941	While the theory that CAS may produce paroxysmal myocardial ischemia is as yet unproved, the recognition of angina-related fixed atherosclerotic obstruction post mortem in 1940s leads to a revised perception that atherosclerosis, rather than CAS, is the chef mechanism of angina pectoris. Furthermore, every patient suffering primarily from angina pectoris without evidence of valvular disease or hypertension has shown old complete occlusion of ≥1 major coronary artery post mortem 8.
1959	Classic Heberden's angina has 2 major features 2, provoked pain by increased work of the heart or disturbance of the mind is relieved by rest or the administration of nitroglycerin an electrocardiography taken during pain shows ST-segment depression in leads I, II, III and V4, without reciprocal elevation
1959	“A variant form of angina pectoris” described by Dr. Myron Prinzmetal 2 has 2 major features, pain at rest or during ordinary activity, but not by effort, is relieved by the administration of nitroglycerin ST-segment elevation with reciprocal ST-segment depression
1959	An experimental intermittent occlusion of a large epicardial coronary artery in 25 dogs performed by Prinzmetal et al. 10 reproduced chest pain and electrocardiographic abnormalities of variant angina, including arrhythmias and non-progressive ST segment elevation in the corresponding leads 10. These changes disappeared and reappeared repeatedly by loosening and tightening the ligature, suggesting that hypertonus of a diseased vessel is probably causing this syndrome. Interestingly, the area of ischemia due to CAS demonstrates systolic ballooning 10.
1962	An occasional angiographic documentation of CAS in a patient with angina and normal electrocardiography at rest is reported 103.
1963	Ergonovine, an ergot alkaloid used to control postpartum uterine bleeding, was found in 1949 to provoke angina, and was proposed in 1963 as a diagnostic test for coronary disease 105.
1972	Contrast-induced CAS, which reproduces chest pain, is demonstrated angiographically in a patient with Prinzmetal angina 85.
1972	The 1st provocative testing using intravenous ergonovine is performed by the Cleveland Clinic 101.
1974	Yasue et al. reports the use of subcutaneous injection of methacholine in provoked CAS 105.
1978	CAS is associated with variable threshold exertional angina 23, 41.
1987	The 1^st^ provocative testing using intracoronary ergonovine is performed by Hackett et al. 104.
1988	The 1st provocative testing using intracoronary acetylcholine is performed by Okumura et al. 106.
1992	Risk factors for CAS were not known until a U.S. study in 1992 demonstrated that smoking was a risk factor for CAS in young women 140.
1993	Smoking is a major risk factor for Japanese CAS patients 141.
2000	CAS-related regional wall motion abnormality, left ventricular dilation and reduced ejection fraction improve 6 months to >1 year after medical treatment, including calcium channel blockers and nitrate/nicorandil 50, 53.
2005	C-reactive protein level is an independent risk factor of CAS in Taiwanese patients 142.

CAS = coronary artery spasm.

**Table 2:** Clinical characteristics and electrocardiographic abnormalities of CAS.

Symptoms	Signs	Electrocardiography
Chest pain, oppressive or stabbing 114, with radiation at rest, at night, during sleep 13, 43, during ordinary activity, or when awakening in the morning 43	Hypotension 10, 13, 57, 65	ST-segment depression without reciprocal change 63 Widespread: diffuse but less severe CAS 22 Isolated in the anteroseptal and anterior leads: CAS of a diagonal or septal branch 22
Waking up from sleep with chest pain 13	Bradycardia (more frequent than tachycardia 43, 57	ST-segment elevation with reciprocal ST-segment depression
Variable threshold exertional chest pain 41	Tachycardia (less frequent than bradycardia) 22	T wave change (inversion, peaking) 22,23
Dyspnea 13, 50	Reversible regional wall motion abnormality, reversible left ventricular dilation, reversible heart failure with reduced ejection fraction 50, 53-56	Peaking T wave followed by ST-segment elevation due to 100% CAS-induced occlusion 22
Cold sweats 13, 17	Dilated cardiomyopathy with atrial fibrillation 54	Pseudonormalization of ST-segment and T-wave 10
Nausea 13	Transient left ventricular wall motion abnormality 143	Taller and broader R wave or disappearance of R wave 10, 65
Dizziness 13	Arrhythmia with or without angina 10, 43, 83	Arrhythmias, mostly ventricular, include sinus tachycardia, sinus bradycardia, sinus arrest with or without junctional escape beats, atrial premature complex, paroxysmal atrial tachycardia, paroxysmal atrial fibrillation 42, 1^st^ degree atrioventricular block 13, 2^nd^ degree (Mobitz I and II) atrioventricular block 13, 70, 3^rd^ degree complete atrioventricular block 13, ventricular premature complex, ventricular bigeminy, ventricular tachycardia, pulseless electrical activity and asystole
Syncope 17, 83	Spasm of digital arteries 42	ventricular fibrillation, including those episodes which revert spontaneously 84, and which require electrical defibrillation
Palpitation 43		Left bundle branch block, incomplete or complete 11, 12, 42, 77
		Right bundle branch block 76
		Decreased S wave magnitude 47
		Negative U wave 47

CAS = coronary artery spasm.

## Abbreviations

CAS: coronary artery spasm
HFrEF: heart failure with reduced ejection fraction
VF: ventricular fibrillation
